# Where is clinical research for radiotherapy going? Cross-sectional comparison of past and contemporary phase III clinical trials

**DOI:** 10.1186/s13014-020-01489-4

**Published:** 2020-02-14

**Authors:** Sunmin Park, Chai Hong Rim, Won Sup Yoon

**Affiliations:** Radiation Oncology, Ansan Hospital, Korea University, 123 Jeokgeum-ro, Danwon-gu, Ansan, Gyeonggi-do 15355 Republic of Korea

**Keywords:** Radiotherapy, Clinical trials, Hypofraction, Stereotactic radiotherapy

## Abstract

**Purpose:**

The features of past and contemporary phase III clinical trials for radiotherapy were reviewed to activate future clinical trials and to advise on actual clinical practice.

**Methods and materials:**

The phase III clinical trials for radiotherapy were searched in the database of ‘ClinicalTrials.gov’ by the U.S. National Institute of Health. Using the staring date, the studies during each period of 4 years were collected for the past (from Jan 2000 to Dec 2003) and contemporary (July 2014 to June 2018) years. For the investigated subjects, the patterns of studies were classified as: Category A, the comparisons of rival radiotherapy protocols; Category B, the comparisons of multidisciplinary approaches; Category C, the investigation of supplementary agents; and Category D, the investigation of optimal partners for concurrent radiotherapy.

**Results:**

The number of studies increased, from 96 past to 158 contemporary studies. The patterns of studies were similar with the mild increase of Category A in the contemporary years (22.9% vs. 29.1%). For the study locations and the funding sources, the Chinese studies (2.1% vs. 34.2%, *P* < 0.001) and the affiliated institutions of researchers (37.5% vs. 72.2%, P < 0.001) markedly increased in the contemporary years from the past Western studies and non-profit organization, respectively. The robust radiation techniques were more usual in the contemporary years (11.5% vs. 44.9%, *P* < 0.001). The fractionation schedule and delivery technique were the common issues in both past and contemporary years of Category A. In Category B, the indications of stereotactic radiotherapy was the rising concern, with eight ongoing studies. Except for the studies of palliative or prophylactic goals and stereotactic radiotherapy, the escape from conventional fraction size was 37.9% (36/95) in the contemporary years with the median fraction size of 2.5 Gy (range 2.05–6.6 Gy) in the comparison with 19.0% (15/79) in the past years (*P* = 0.006).

**Conclusions:**

To activate the clinical trials for radiotherapy, the funding sources would be diversified, including industrial support. Hypofractionated schedules using robust techniques could be preemptively considered in actual clinical practice.

## Introduction

Radiotherapy has had a long-term history of over a hundred years of treating malignant cancer after X-rays and radium were discovered at the end of the nineteenth century. Initially, radium and low-energy machines were used for the easily accessible tumors and radiotherapy began to expand the field to all malignant cancers thanks to the generalization of mega-voltage linear accelerators [1]. Currently, the radiotherapy usage rates as the first course of treatment reached about 31% in US 2014 statistics [2]. However, for better clinical outcomes through qualified radiotherapy, a radiation oncologist must understand the place of radiotherapy and cooperate with the surgical and medical oncologists in this era of multidisciplinary approaches.

Clinical trials systematize the usefulness of individual clinical experience and distinguish the values of specific treatments. The well-designed randomized controlled clinical trials can establish the evidence-based medicine to guide the standard management and to suggest future strategies. Actually, the phase III clinical trials which were completed before a decade would construct the present clinical guideline in consideration of the mature period. In addition, through the overview of the recent clinical trials, the emerging issues could be well identified. For optimal radiotherapy, there is no better method than to look back at the implemented and implementing clinical trials with radiotherapy.

Hence, we reviewed the features of phase III clinical trials in the past and contemporary radiotherapy. Consequently, the radiation oncologists could figure out the context of change and existing problems, and get advice on actual clinical practice and future clinical trials. In addition, the directions to further activate clinical researches could be discussed in radiation oncology.

## Methods

For searching for information on clinical trials, we used the database of ‘ClinicalTrials.gov’ by the U.S. National Institute of Health. The clinical trials of phase III including the term “Radiotherapy” were searched for in terms of all study statuses (recruiting/ enrolling by invitation/ active, not recruiting/ suspended/ terminated/ completed/ withdrawn/ unknown status) except for the status of “not yet recruiting”. The starting date of the study was limited from Jan 2000 to Dec 2003 and from July 2014 to June 2018. The eligible criteria were studies in which (1) radiotherapy had an obvious role for the therapeutic outcomes, (2) radiotherapy was done for a malignant cancer including borderline malignancies, and (3) external beam radiotherapy was applied in any arm. The exclusion criteria were studies in which (1) the subject was hematologic or lymphatic malignancies or only children, (2) the stratification was done after performing radiotherapy, (3) the effectiveness of neoadjuvant or consolidative management that did not involve radiotherapy was investigated without the change of radiotherapy protocol in all allocated arms, and (4) the details of hormonal therapy, such as drug combination, duration and timing, were investigated in breast and prostate cancer.

First, to know the patterns during each period of 4 years, the past years (Jan 2000 to Dec 2003) and the contemporary years (July 2014 to June 2018) studies were divided according to the start date of the study. Second, the studies were classified with four categories in terms of the investigating subjects as below.

- Category A: The studies to compare rival radiotherapy protocols, (e.g. treatment schedule, radiation field, or techniques),
- Category B: The studies to compare the standard therapy and new ones in multidisciplinary approaches,
- Category C: The studies to investigate supplementary agents (management) to support the therapeutic effectiveness and tolerability of radiotherapy, and
- Category D: The studies to investigate optimal partners of pharmaceutical agents or procedures with radiotherapy.

The information on protocol number, study status, disease conditions, radiotherapy aim, the endpoints, sponsor/collaborators, study location, and the details of radiotherapy, surgery, and pharmaceutical agents was collected from the web page of “ClinicalTrials”. If the details of treatments for each study were insufficient, the open information was collected from the web sites “Pubmed” and “Google” using protocol number and other ID of trials.

The main end-points of this study were (1) to measure the volume of clinical trials regarding radiotherapy, (2) to observe the changes of funding sources and study locations, (3) to consider the change of radiation schedule and fraction size, and (4) to check the application of the state-of-the-art techniques. A Chi-square test was conducted to compare the difference of the past and contemporary years. A two-sided *p* < 0.05 was considered significant. SPSS 20.0 (IBM SPSS, Inc., Chicago, IL) was used for the analysis.

## Results

**Fig. 1:**
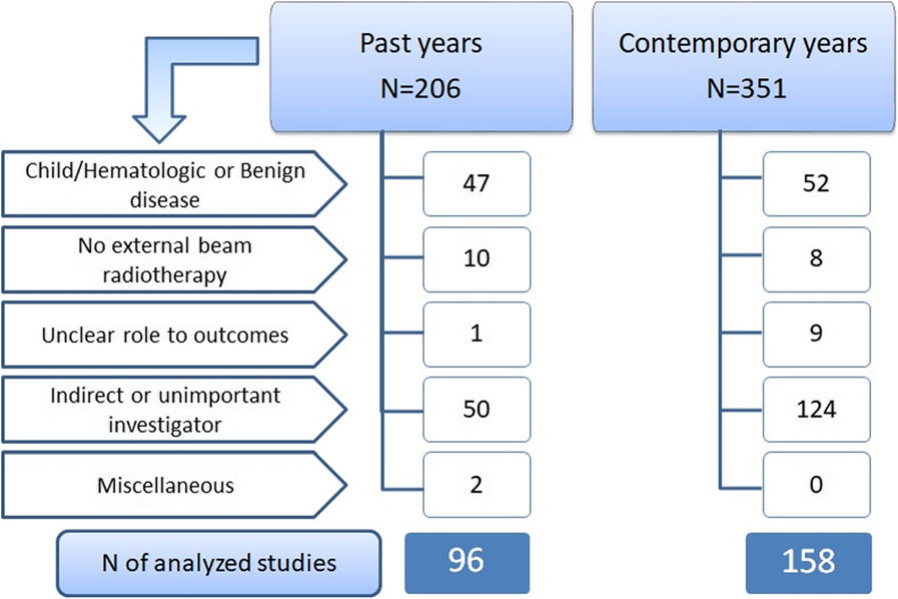
Flow of selection process from all phase III studies with the keyword “radiotherapy” to eligible studies

Of the total of 206 past and 351 contemporary studies, 96 and 158 studies satisfied our eligibility criteria for our studies, respectively. (Figure 1) The number of phase III clinical trials regarding radiotherapy increased by 64.6% in the contemporary years. While Category A increased by 6.2% in the contemporary years in comparison with the past years, however, the difference was not remarkable (*P* = 0.309). One and three studies in the past and contemporary years had to be included in both categories, because they were designed by the 2 X 2 fractions model, and all four studies were associated with category A. The primary radiotherapy was intended to cure the actual tumors in 58.3 and 63.9% of the past and contemporary studies, respectively (*P* = 0.299). The studies administering concurrent chemotherapy were used in the past years of 54.1% and contemporary years of 63.3% (*P* = 0.350). Whereas the past studies was done in Western areas and supported by non-profit organizations, the studies from China and affiliated institutions of the researcher markedly increased in the contemporary years (both *P* < 0.001). The weak industrial supports were unchanged in the past (7.3%) and contemporary (8.9%) years. For the endpoints, the toxicity was more commonly observed in contemporary years (*P* = 0.003) (Table 1).

**Table 1:** Studies’ characteristics in the past and contemporary years

	Past years (Jan 2000 – Dec 2004, *N* = 96)	Contemporary years (July 2014 – June 2018, *N* = 158)	*P* value
Category^a^			
A	22 (22.9%)	46 (29.1%)	0.309
B	39 (40.6%)	64 (40.5%)	0.807
C	18 (18.8%)	22 (13.9%)	0.306
D	18 (18.8%)	29 (18.4%)	0.937
Aim of radiotherapy^a^			
Adjuvant	27	43	0.875
Definite	56	82	0.299
Neoadjuvant	11	19	0.892
Palliative	7	21	0.139
Prophylactic	2	4	
Any	1	1	
Disease status			
Non-: Metastatic: Any	86: 6: 4	133: 23: 2	0.065**
Naïve: Recurrent: Any	88: 1: 7	135: 2: 21	1.000**
Sponsors/Collaborators^a^			
Non-Profitable organization	67	47	< 0.001
Industry	8	14	0.885
Institution of researcher	36	114	< 0.001
Locations			< 0.001
Western: China: World-wide: Others	82: 2: 5: 7	76: 54: 10: 18	
Participant Institutions			0.770
Single: Multiple	27: 69	41: 117	
Concurrent administration of drugs			0.350**
Yes: No: Not specified	53: 40: 3	100: 58: 0	
Robust delivery technique^a^	11	71	< 0.001
SBRT	3	22	
IMRT	8	48	
Proton	0	3	
Endpoints^a^			
Survival	76	129	0.627
Tumor response	33	33	0.017
Toxicity	52	114	0.003
Quality of life	40	70	0.681

^a^duplicated with other sub-items

**Chi-square tests were conducted excluding “any” or “non-specified” items

Category A: to compare rival radiotherapy protocols, B: to compare the strategies in multidisciplinary approaches, C: to investigate supplementary agents for radiotherapy and D: to investigate optimal partners with radiotherapy

Western area included USA, Canada, European countries, Australia and New Zealand

*SBRT* Stereotactic body radiotherapy, *IMRT* Intensity modulated radiotherapy

**Fig. 2:**
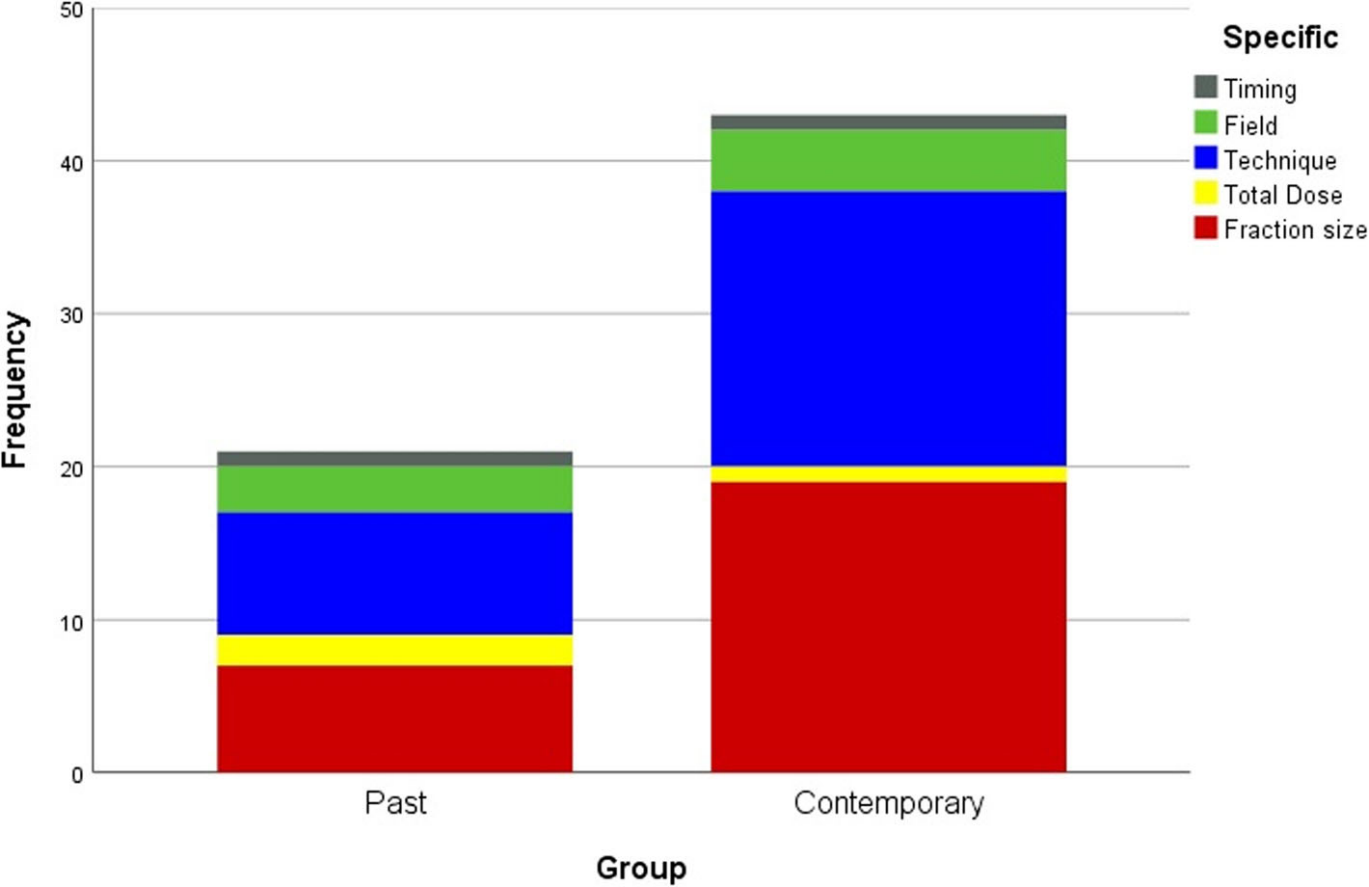
The number of studies that compared rival radiotherapy protocols doubled, from 22 in past years to 46 in contemporary years. The two main issues were fraction size and radiation technique in both the past and contemporary years

The contemporary studies in Category A were concerned with the fractionation schedule in 43.5% (20/46) of cases without the significant change of detailed patterns. (Fig. 2) The applications of hypofraction schedules was expanded from rectal and prostate cancers of the past years to breast, lung, esophagus, and head and neck of the contemporary years, and the median fraction size of the experimental group was 2.66 Gy (range 2.05–5 Gy) if one stereotactic body radiotherapy (SBRT) was excluded. However, it was not observed to use hyperfraction schedules in the contemporary years. Meanwhile, the dose escalation of prostate cancer was investigated in the past years; the dose de-escalation of human papillomavirus (HPV) positive head and neck cancer was examined in contemporary years. The studies regarding dose prescription advised by positron emission tomography (PET) were noticeable in lung, head and neck and cervical cancer (Supplementary 1).

Of the category B, to decide the optimal strategies of multidisciplinary approaches, a few changes were found. In the past years, the main concerns were concurrent chemoradiotherapy (CCRT) vs. radiotherapy or chemotherapy alone (13 studies) and additional consolidation therapy after radiotherapy (7 studies). Currently, the rising concerns were additional radiotherapy using SBRT (8 studies) in oligometastases, brain metastases, and hepatocellular carcinoma, and the comparison of adjuvant vs. neoadjuvant therapies (6 studies) in soft tissues, stomach, rectal, and penile cancer.

For category C, the radio-sensitizers, general tolerability or pain and specific toxicity were equally examined in the past years. The contemporary concerns were intensified in the areas of acute toxicity, such as mucositis, skin reaction and urinary symptom. For Category D, new pharmaceutical agents were actively reflected in the contemporary years. Whereas most studies were on the traditional chemo-agents (77.8%, 14/18) in the past years, the studies administering targeted agents, immunotherapy and antivirals (41.4%, 12/29) caught up much of the chemo-agents in contemporary years (Table 2).

**Table 2:** Investigations’ characteristics in category C and D

	Past years (Jan 2000 – Dec 2004)	Contemporary years (July 2014 – June 2018)	*P* value
Category C			0.364
Sensitizer	6	4	
General tolerability or Pain	6	6	
Specific toxicity	6	12	
- Skin reaction	- 1	- 4	
- Oral mucositis	- 3	- 7	
- Xerostomia	- 2	- 0	
- Urinary symptom	- 0	- 1	
Category D			0.002
Cytotoxic drug	14	17	
New drug	2	12	
- Targeted agent	- 2	- 8	
- Immunotherapy	- 0	- 3	
- Antivirals	- 0	- 1	
Surgery	2	0	

**Fig. 3:**
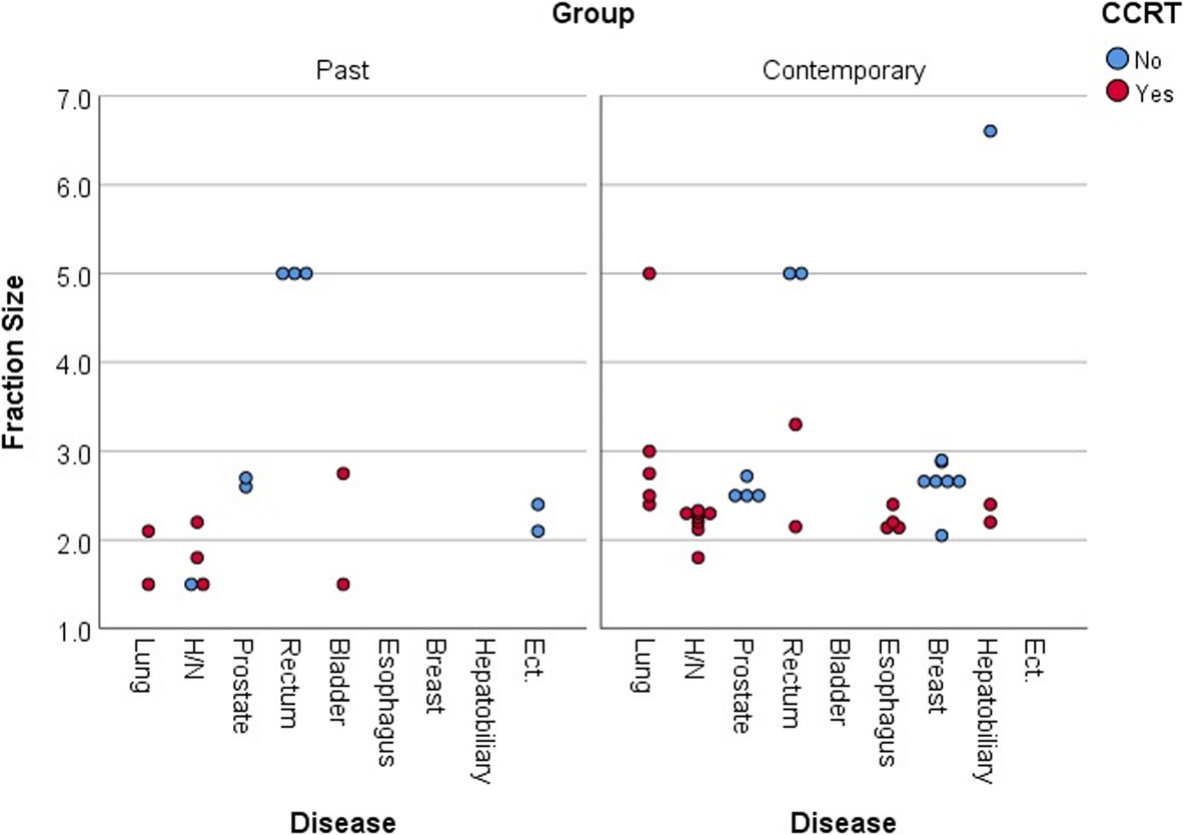
Studies using hyper- or hypo-fractional radiotherapy. Hyperfraction schedules for lung, head and neck, and bladder cancer were tried in past years (left panel), but the interests decreased in contemporary year (right panel). Hypofraction schedules were newly tried for breast and hepatobiliary cancer in contemporary years. * The studies applying a hyperfraction schedule were indicated below the line of 2.0 Gy

After excluding the studies with palliative or prophylactic aims and applying SBRTs, 79 and 95 studies of the past and contemporary years, respectively, addressed their fraction schedule of radiotherapy in protocols. The escape from conventional daily fraction size of 1.8–2 Gy was 19.0% (15/79, 5 hyperfraction regimen) in the past years and 37.9% (36/95, 1 hyperfraction regimen) in the contemporary years (*P* = 0.006). In terms of CCRT protocol, 14.6% (7/48, 5 hyperfraction regimen) and 27.3% (21/77, 1 hyperfraction regimen) of studies used a daily fraction size higher than 2 Gy in the past and contemporary years, respectively (*P* = 0.098). The median fraction size of hypofraction was 2.5 Gy (range 2.05–6.6 Gy) and 2.3 Gy (range 2.12–5.0 Gy) in whole and CCRT studies in the contemporary years, respectively (Fig. 3).

## Discussion

Although the phase III studies concerning radiotherapy were abundantly registered, their increasing rates were not surprising in comparison with the growth rates of all fields concerning cancer. In the same periods of the past and contemporary years, the registered phase III clinical trials to be searched for with the keyword of “cancer” doubled, from 827 studies to 1674 studies on the database of ‘ClinicalTrials.gov’. Of course, that is related to the growing market for pharmaceutical agents in malignancy treatment, and the industrial sponsors strongly supported approximately a third (286/827) of the studies in past years and half of the studies (757/1674) in the contemporary years. On previous report to analyze oncologic trials regardless of the phases of study during recent 10 years, the radiotherapy trials consisted of only 5.3% of whole trials and received a week industrial support of 5.8% [3].

How can the studies about radiotherapy be more actively performed? The phase III studies of 2 X 2 fractional stratification, we guess, could be a good model. For example, RTOG 0617 studies examined total radiation dose and usefulness of cetuximab in advanced-stage lung cancer [4], and four studies in our review, including NCT00024349 (CRC-BC2001) for bladder cancer, applied this stratification [5, 6]. Of course, these kinds of protocols need more eligible numbers to inhibit the under-power of statistics and thus, they need more time and cooperation between physicians to publish the final outcomes, in addition to the effort to make well-designed protocols. However, it is more economical to reduce the duplicated labors and costs of clinical trials if the eligible condition to investigate is similar. From this viewpoint, the communication of radiation oncologists and other oncologists could be necessary to clarify the specific details for radiotherapy and multidisciplinary management earlier. The industrial funds to plan new pharmaceutical agents could also be indirectly used for radiotherapy.

The expansion of clinical trials to non-Western countries could be welcome. It can increase the number of studies and give more clinical information for the more frequently developed malignancies in non-Western countries. However, to insure the quality of studies, it is necessary to transfer the know-how and settle for an efficient system in these emerging locations. It is a typical model that the alliance of National Clinical Trials Network could insure the quality of studies for radiotherapy through the structure name libraries and software tools and templates in the US [7].

Thanks to intensity modulated radiotherapy (IMRT) techniques and the concept of simultaneous integrated boost to intensify the radiation dose on the restricted local area, a hypofractionated schedule would be the inevitable trend for radiotherapy, making it more comfortable for patients by reducing the treatment period while showing equivalent clinical outcomes. It is notable that the median 2.5 Gy was applied in approximately one third of the contemporary trials. The SBRT technique broadened its areas from brain metastases to early-stage lung cancer, oligometastases, and hepatocellular carcinoma. Through the robust advance of linear accelerators [8], liniac-based stereotactic radiosurgery rapidly disseminated in brain metastases in the US [9]. There were a few reports about cost effectiveness that favored the SBRT over open surgery in brain metastases and lobectomy in early lung cancer [10, 11]. The clinical outcomes were prospective in a phase I/II trial of hepatocellular carcinoma [12] and an initial trial of oligometastases in prostate cancer [13], and the relevant studies using SBRT could be continued. Additionally, the prescription guidance by PET is interesting for achieving a personalized hypofraction schedule. It was feasible in the initial reports of head and neck cancer, non-small-cell lung cancer, and esophageal cancer [14–17].

The phase III clinical trials were designed on the basis of the positive outcomes of early phase studies or credible observations. If the progress of experimental investigations is active both in quality and in quantity, there would be some portions to preemptively consider those practice case by case. Surely, other experts suggested that the cost-effectiveness of new strategy preferentially is assessed [18]. We thought that the above mentioned trends of hypofraction schedule using IMRT, SBRT and the collaboration with novel image technologies could be major candidates.

The therapeutic ratio for late toxicity could be an interesting viewpoint in the comparison of the past and contemporary studies. Meanwhile, the radiation protectors of amifostine and supplementary agents of salagen used for reducing late toxicity, such as xerostomia, the hippocampal-sparing brain radiotherapy using the IMRT technique [19], and the dose de-escalation in HPV patients using biomarkers [20], were actively tried in the contemporary years. The supplementary agents focused on acute and subacute toxicity of oral mucosa and skin. One may also wonder whether the synergistic effects of the combination of radiotherapy and immunotherapy could replace the cytotoxic chemotherapy [21].

There were a few limitations of this review. First, researchers reported on their studies freely on the platform of “ClinicalTrials.gov” and the records were largely faithful. However, there was some missing information we wanted to collect, especially in the past years. Second, there was a possibility that we missed studies, because we used only the platform of “clinical trial,” although it is the best known one in the world. Last, we observed the studies by the cross-sectional method because of the bulk loading to review all clinical trials from 2000 to 2018. Therefore, it would be sufficient to see the landscape of clinical trials regarding radiotherapy and prepare for future studies.

## Conclusion

The number of clinical trials consistently increased in non-Western area, especially. To more activate the clinical trials for radiotherapy, it is necessary that the funding sources should be diversified, including industrial support. Hypofractionated schedules using robust techniques which were investigated in the contemporary years could be preemptively considered in actual clinical practice for various kinds of cancer. Radiation oncologists have to understand the trends of clinical trials for radiotherapy and try the next well-designed clinical trials. Keeping in mind the place of radiotherapy in multidisciplinary approaches overall, the cooperation with medical and surgical oncologists would effectively promote better clinical trials and establish the evidence for radiotherapy sooner.

## Supplementary information

**Additional file 1:Supplementary 1.** The details of investigations in category A (comparison of rival radiotherapy protocols).

## Data Availability

The data that support the findings of this study are available in http://www.ClinicalTrials.gov.

## Abbreviations

CCRT: Concurrent Chemoradiotherapy
HPV: Human Papillomavirus
IMRT: Intensity Modulated Radiotherapy
PET: Positron Emission Tomography
SBRT: Stereotactic Body Radiotherapy
