# Supplementary material for: Where is clinical research for radiotherapy going? Cross-sectional comparison of past and contemporary phase III clinical trials
Source: Radiat Oncol. 2020 Feb 14;15:36. doi: 10.1186/s13014-020-01489-4 (PMC7023759; doi:10.1186/s13014-020-01489-4)
Supplement: Supplementary file 1 — Additional file 1:Supplementary 1. The details of investigations in category A (comparison of rival radiotherapy protocols). [file 13014_2020_1489_MOESM1_ESM.docx]

Supplementary 1. The details of investigations in category A (comparison of rival radiotherapy protocols)

|  | Past years  (Jan 2000 – Dec 2004, N =22) | Contemporary years  (July 2014 – June 2018, N =46) | P value |
| --- | --- | --- | --- |
| Fractionation schedule  - Hypofraction  - Comparison of rival SIB  - Hyperfraction | 7  Prostate (2)  Rectum (2)  Head and Neck (2)  Prostate | 20  Breast (5)  Esophagus  Lung (3)  Prostate (2)  Rectum (3)  Spine M  Breast  Esophagus  Head and Neck  Lung  Rectum | 0.358 |
| Total Dose | 2  Prostate (escalation, 2) | 2  Head and Neck (de-escalation, 2) |  |
| Timing | 1  Lung | 1  Lung |  |
| Field | 4  Breast  Bladder  Lung  Unknown HN | 4  Breast  Cervix  Nasopharynx  Prostate |  |
| Delivery technique | 8  Brain (Fixation method)  Breast (IMRT)  Breast (IORT)  Endometrium (ICR)  Prostate (BRT+EBRT)  Prostate (IMRT)  Brain M (SRS+WBRT, 2) | 19  Brain (IMRT, 2)  Brain (IORT)  Brain (PET guidance)  Cervix (BRT)  Cervix (PET guidance)  HCC (Proton)  Lung (PET guidance)  Prostate (BRT)  Prostate (SBRT)  Rectum (ICR)  Brain M (IMRT)  Brain M (SRS(SBRT) 4)  Spine M (IORT)  Spine M (SBRT, 2) | 0.697 |

* (#number): Total number of studies if similar study was over one.

SIB; Simultaneous integrated boost, M; Metastases, IMRT; Intensity modulated radiotherapy, IORT; Intraoperative radiotherapy, ICR; Intracavitary radiotherapy, BRT; Brachytherapy, EBRT; External beam radiotherapy, SRS; Stereotactic radiosurgery, WBRT; whole brain radiotherapy, PET; Positron emission tomography, HCC; Hepatocellular carcinoma, SBRT; Stereotactic body radiotherapy
